# Complex aetiology of an apparently Mendelian form of Mental Retardation

**DOI:** 10.1186/1471-2350-9-6

**Published:** 2008-02-06

**Authors:** Ana Beleza-Meireles, Ingrid Kockum, Qiu-Ping Yuan, Simone Picelli, Lennart Wetterberg, Karl-Henrik Gustavson, Martin Schalling

**Affiliations:** 1 Department of Molecular Medicine and Surgery, Karolinska Institutet, Stockholm, Sweden; 2 Institute of Psychiatry, King's College London, UK; 3 Department of Clinical Neurosciences, Karolinska Institutet, Stockholm, Sweden; 4 Department of Clinical Genetics, Rudbeck Laboratory, Uppsala University Hospital, Uppsala, Sweden

## Abstract

**Background:**

Mental Retardation is a common heterogeneous neurodevelopment condition, which causes are still largely elusive. It has been suggested that half of the phenotypic variation of intelligence is explained by genetic variation. And genetic or inherited factors indeed account for most of the cases of mental retardation with an identifiable cause. However, only a few autosomal genes have been mapped and identified to date. In this report, the genetic causes for an apparently recessive form of mental retardation, in a large nordern swedish pedigree, are investigated.

**Methods:**

After extensive evaluation of the patients, which ruled out recognizable patterns of malformation and excluded known causes of MR, a comprehensive genome-wide linkage analysis, with 500 microsatellite markers, was performed in 24 members of this family. Additionally, a genome-wide copy number analysis, using an affimetrix 250 K SNP chip, was performed in this pedigree.

**Results:**

No significant LOD score was found with either parametric and non-parametric linkage analysis. The highest scores are located at chromosomes 13, 15 and 17. Genome-wide copy number analysis identified no clear cause for the disorder; but rather, several variants were present in the family members, irrespective of their affected status.

**Conclusion:**

These results suggest that mental retardation in this family, unlikely what was expected, has a heterogeneous aetiology; and that several lower effect genes variants might be involved. To demonstrate such effects, our family may be too small. This study also indicates that the ascertainment of the cause of MR may be challenging, and that a complex aetiology may be present even within a pedigree, constituting an additional obstacle for genetic counselling. Variants in genes involved in molecular mechanisms of cellular plasticity, in genes involved in the development of underlying neural architectures, and in genes involved in neurodevelopment and in the ongoing function of terminally differentiated neurons may underlie the phenotypic variation of intelligence and explain instances of intellectual impairment.

## Background

Mental retardation (MR) is characterized by severe limitations in intellectual functioning and in adaptive behaviour, as expressed in conceptual, social and practical adaptive skills and with onset before the age of 18 [1,2]. This disability originates as a consequence of abnormal brain development and impaired cognition. MR, as classified by the DSM-IV, affects approximately 2–3% of children and young adults, being one of the frequent neuropsychiatric disorders [1,2]. Non-syndromic mental retardation (NSMR) is the diagnosis of exclusion in mentally retarded individuals who do not have dimorphic features, or neurological abnormalities.

We are far from understanding the biology of MR, due to its extraordinary heterogeneity. Despite common, its causes remain unexplained for more than 50% of patients, limiting the efficiency of genetic counselling, detection of carriers, prenatal diagnosis in the affected families and constituting one of the most important unresolved problems in clinical genetics [3].

In the cases in which the aetiology is known, genetic or inherited aetiologies are implicated in a considerable fraction of the cases [3,4]. These include chromosomal rearrangements that result mainly in pathogenic gene dosage effect, alteration of the imprinting in specific genes or genomic regions and dysfunction of single genes. A recent analysis of the Online Mendelian Inheritance in Man (OMIM) database indicated that about 280 unrelated chromosomal aberrations and single-gene mutations involve or cause mental retardation [4]. These genes are distributed over a broad range of functions, including enzymes, mediators of signal transduction and transcription regulation, binding proteins, and transporters [4-7]. Numerous aspects of neuronal differentiation and synaptic plasticity, synaptic vesicle cycling and gene expression regulation are considered to be important in the aetiology of MR. Hundreds more MR genes remain to be identified.

A monogenic mode of transmission may account for nearly one-fourth of these cases [7-9]. However only a few autosomal genes have been mapped and identified to date: The PRSS12 gene on chromosome 4q25-q26 in one Algerian family (MRT1; MIM #249500), the CRBN gene on 3p26.2 in one large American family with German ancestry (MRT2A; MIM #607417), the CC2D1A gene on 19p13.12 in one large Arab-Israeli family (MRT3; MIM #608443) and the ionotropic glutamate receptor 6 gene (GRIK2; MIM # 138244) on 6q16.1-q21, which is associated with autosomal recessive mental retardation in a large, consanguineous Iranian family [10-13]. The existence of large family pedigrees with non-syndromic, autosomal forms of MR can be important tools to the use of genetic linkage analysis to identify causative genes and may thus shed light on the biology of MR.

Autosomal recessive diseases are common in isolated populations, mostly as a result of the high rate of consanguinity [14]. In this report, the genetic causes for an apparently recessive form of non-syndromic mental retardation, in a large nordern swedish pedigree, are investigated. This study is a follow up of a prevoius report by Gustavson K-H, et al, 1986 [15].

## Methods

An Ethics Committee at the Karolinska Institute has approved the protocols for this research project. And it conforms to the provisions of the Declaration of Helsinki in 1995 (as revised in Edinburgh 2000). Subject, and/or their parents, gave informed consent and patient anonymity has been preserved.

### Description of the family and the geographic region

Patient material was initially collected between, 1946 and 1983, from a large family living in a North Swedish isolate. This area, of about 5000 Km^2^, is located at the Swedish-Finnish border, in the fork of 2 large rivers, the Muonio and the Torne, and includes 3 parishes: Pajala, Muonionalusta and Junosuando. Only after the end of the 16^th ^century, was some settlements registered by north Finnish families. By the beginning of the 19^th ^century, the population was about 2,000, many of them related to the first settlers. The particular geography and demography contributed to the creation of an isolate, where a large number of consanguineous marriages occurred.

### The family

**Figure 1:**
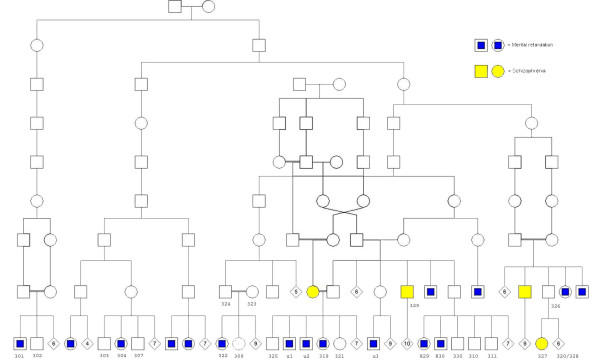
**Northern Swedish Pedigree**. The study includes 24 individuals from a large family, partially overlapping in more distant generations, including 9 mild mentally retarded individuals (301, 304, 322, u1, u2, 319, u3, 829 and 830), one individual affected with schizophrenia and mild mental retardation (329), 13 non affected individuals (302, 303, 307, 323, 324, 309, 321, 330, 310, 311, 320, 326, 328) and one individual with schizophrenia (327). Individual 325 was not included due to insuficient phenotypic and biological data.

The present study included 24 individuals from a large family, partially overlapping in more distant generations. The pedigree can be seen in figure 1, and the studied individuals are the ones marked with a numeric or alfa-numeric code, and includes 9 mild mentally retarded individuals (301, 304, 322, u1, u2, 319, u3, 829 and 830), one individual affected with schizophrenia and mild mental retardation (329), 13 non affected individuals (302, 303, 307, 323, 324, 309, 321, 330, 310, 311, 320, 326, 328) and one individual with schizophrenia (327). Individual 325 was not included due to insuficient phenotypic and biological data. Extensive clinical, biochemical, imaging and genetic examinations have been carried out. The clinical evaluation was extensive but inconsistent. The following paragraphs present a concise characterization of the patients.

### History/Physical examination

All the mentally retarded individualswere physically examined by one of the authors, K-H G, specialist in pediatrics and clinical genetics. The MR patients presented no major malformation and no recognizable pattern of malformations was identified. Non-consistent minor signs were found in some individuals; eight of the physically examined patients were short and had a picnic, but not obese, body type. Psychological evaluation revealed an overall low level of intelligence (IQ around 50), with lower ability of logical thinking. Attention deficits are present and characterized by a focus towards an apparent part of an event and to an episode's different parts rather than the correlation between them. Among themselves they have reached diverse skills spatial and perceptually. Otherwise, the patients are outgoing and with good social interaction.

### Cytogenetics/Molecular cytogenetics

Five of the patients underwent karotyping and subtelomeric rearrangement analysis at the Clinical Genetic Departments of the Uppsala University Hospital and of the Karolinska Hospital. Major chromosomic inbalances were ruled out by QM, R and C-banding karyotyping. Subtelomeric cryptic rearrangements by fluorescence in situ hybridization (FISH) probes from the subtelomeric regions of all chromosome arms were not found [16]. Repeat expansion detection (RED) was performed, and no expanded trinucleotide repeat was found [17]. Screening for Fragile X was performed by standard procedures in the index patient, and no abnormality was found.

### Metabolic workup

Despite the absence of specific signs of a metabolic disorder, a metabolic workup was performed, including the general assessment of acid/base balance, plasma and urine amino and organic acid studies, thyroid screening tests, lysosomal enzyme analysis, plasma and urine carnitine analyses, plasma very long chain fatty acids, was performed showing no consistent abnormality. Three patients presented higher levels of hydroxyhippuric acid in urine.

### Neuroimaging studies

Four of the patients were admitted to the Department of Psychiatry, St. Göran's Hospital, Stockholm. CT scans of the brain showed widened subarachnoidal space over cerebellum in one patient, general brain atrophy in one other case, widened ventricular system in another case; the three cases presented structural abnormalities indicative of brain atrophy.

### Experimental setup

Peripheral blood samples were collected and the genomic DNA was extracted by standard procedures. Whole-genome screening was performed on the available 24 family members, by typing 500 evenly distributed polymorphic microsatellite markers. The reactions were conducted by deCODE Genetics in Reykjavik, Iceland, upon delivery of the DNA samples. In the present framework marker set, the average spacing between markers was approximately 10 cM.

Before the statistical analysis, allele frequencies were estimated from all genotyped individuals using the zGenStat1.126 program (H. Zazzi, unpubl. data). All genotyped markers were checked for Mendelian incompatibilities using zGenstat1.126 program and with PedCheck program [18]. Either the incompatibility was resolved unambiguously, or individuals were excluded from the linkage analysis at that locus. To identify markers with allele dropout or other problems, the expected number of homozygous individuals was calculated based on the estimated allele frequencies and compared with the observed numbers of homozygotes using the Pearson chi-squared test with zGenStat1.126 software (H. Zazzi, unpubl. data). Any marker deviating significantly from expected homozygosity frequency (P < 0.001) was removed from the analysis. This resulted in the removal of data from two markers. An estimate contribution of affected and unaffected offspring to the LOD score revealed a predicted LOD of 2.7 to 3.9 in a recessive model, and 5.7 to 7.9 in a dominant model, depending on the penetrance.

Single point and multipoint linkage analysis, for both linear and exponential models, was initially performed using non-parametric affected relative-pair-based methods. The linkage analysis, including allele sharing LOD scores, NPL scores and haplotyping, were obtained using the program ALLEGRO version 1.2. using the Allegro software [19]. Non-parametric P-values were interpreted according to Lander and Kruglyak [20], as summarized by Nyholt [21]. For multi-point linkage analysis, the LOD ≥ 2.19 corresponding to a *P*-value of < 7.4 × 10^4 ^is regarded as suggestive linkage.

Additionally, we maximized the LOD scores with respect to the disease model parameters by performing multipoint parametric linkage analysis and haplotype analysis with the program GENEHUNTER-MODSCORE [22] with standard criteria. This software explores the disease-model parameter space in an efficient way. It can handle autosomal or pseudoautosomal loci. It is also capable of taking imprinting into account in a parametric linkage analysis.

SimWalk2 version 2.83 [23] was then used to carry out multipoint parametric linkage analysis and haplotype analysis. This program can analyze large pedigrees by using the Markov chain Monte Carlo and simulated annealing algorithms to compute location scores which are directly comparable to multipoint LOD scores and are presented in log10 units. Single point analysis, using the MLINK program [24], was performed with the linked haplotype as disease allele and any other haplotype as wild type allele. Autosomal dominant and autosomal recessive modes of inheritance were assumed with 80% penetrance and phenocopy rates of 1–10%. Marker positions were obtained from the Marshfield and the Généthon genetic maps [25].

Copy number analysis was performed in the individuals 301, 304, 322, u1, u2, 319, u3, 829, 830, 302, 303, 307, 323, 324, 309, 321, 330, 310 and 311. The individuals 320, 326, 327 and 328 were not included as they were not considered to be very informative. For this procedure we used a high-density oligonucleotide microarrays developed by Affymetrix. Array experiments were performed according to the Affymetrix GeneChip Mapping 250 K array standard protocol (Affymetrix Inc., Santa Clara, CA, USA) at the Uppsala Array Platform, Sweden. We obtained a call rate of approximately 100%. The physical position of all SNPs was mapped according to the latest genome assembly at the UCSC Genome Browser. The analysis was performed with the Chromosome Copy Number Analysis Tool 4.0 (CNAT 4.0), Affymetrix, which identifies genome-wide chromosomal gains and losses and loss of heterozygosity (LOH) [26]. The copy number state was calculated with a smoothing factor of 0.1 Mb, compared to a normal population of about 100 individuals from the HapMap.

## Results

### Microsatellite analysis

**Figure 2:**
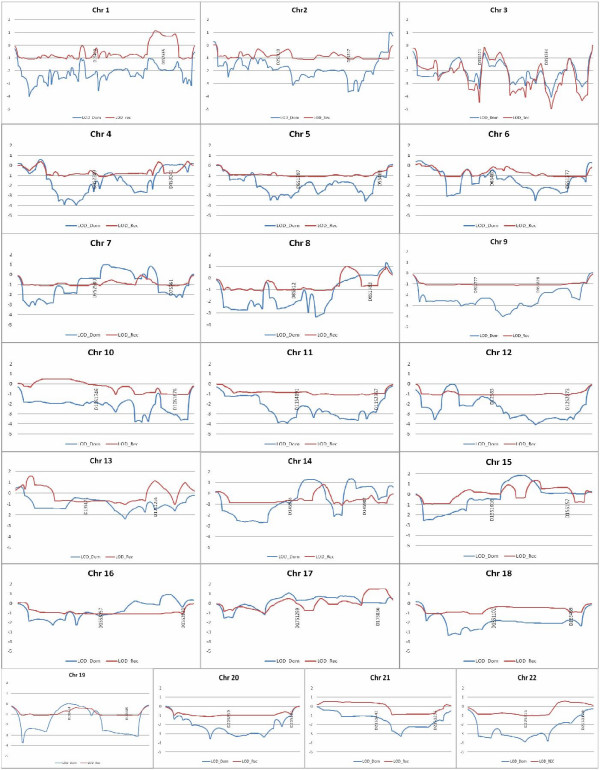
**Whole genome linkage analysis with 500 polymorphic microsatellite markers**. The results of parametric linkage analysis with SimWalk. In blue, the dominant model; in pink, the recessive model.

Parametric linkage analysis, with SimWalk (results in figure 2), testing both recessive and dominant models, assuming 80% penetrance and 1–10% phenocopies, did not identify any LOD higher than 2. The higher peaks were located at:

• **Chromosome 1**, with a location score of 1.14 at 203.99 cM (**1q32.1**) with a recessive model;

• **Chromosome 7**, with a location score of 1.04 at 89.95 cM (**7q21.11**) with a dominant model;

• **Chromosome 8**, with a location score of 1.3 at 166.47cM (**8q24.3**) with a dominant model;

• **Chromosome 13**, with a location score of 1.58 at 5.18 cM (**13q12.11**) and of 1.12 at 100.30 cM (**13q33.1**) with a recessive model;

• **Chromosome 14**, with a location score of 1.3 at 65.88 cM (**14q23.3**) and of 1.34 at 95.71 cM (**14q32.12**) with a dominant model;

• **Chromosome 15**, with a location score of 1.8 at 80.43 cM (**15q24.3**) with a dominant model and of 1.4 at 97.75 cM (**15q26.1**) with a recessive model; and

• **Chromosome 17 **with a location score of 1.1 at 66.89 cM (**17q12**) with a dominant model and of 1.54 at 132.67 cM (**17q25.3**) with a recessive model.

Additional analysis with GENEHUNTER-MODSCORE [see Additional file 1] shows no significant score and the higher peaks (between 1.5 and 2) locate to:

• **Chromosome 3**, with a MOD of 1.64 at 12.37 cM (**3p26.02**);

• **Chromosome 4**, with a MOD of 1.704 at 204.7 cM (**4q35.2**);

• **Chromosome 13**, with a MOD of 1.87 at 4.32 cM (**13q12.11**) and of 1.706 at 27.8 cM (**13q12.3**);

• **Chromosome 15**, with a MOD of 1.95–1.97 between 64.33 cM (**15q22.2**) and 80.43 (**15q24.1**) cM;

• **Chromosome 17, **with a MOD of 1.7 between 126.6 cM (**17q25.3**) and 132.7 (**17q25.3**) cM;

• **Chromosome 18, **with a MOD of 1.5 between 82.41 cM (**18q21.32**) and 88.85 cM (**18q21.33**); and

• **Chromosome 21**, with a MOD of 1.6 from 0 cM to 1.53 cM (**21q11.2**).

The results of the non-parametric linkage analysis with ALLEGRO revealed no significant LOD score and all LOD scores were lower than 0.5.

### Copy number analysis

No clear cause for the disorder was identified, meaning that no copy number variant was identified only in the affected individuals (Results in tables 1 and 2; and Additional files 2, 3, 4). But rather, several copy number variations were present in all the family members, with no pattern of distribution, and irrespective of their affected status, as compared with a normal population of about 100 individuals from the HapMap project.

**Table 1:** Copy number variants encountered in the Northern Swedish family: Aberrations found in the different individuals; several copy number variations were present in all the family members, with no pattern of distribution, and irrespective of their affected status, as compared with a normal population of about 100 individuals from the HapMap project. Aberrations found only once, and in healthy individuals, and aberrations apparently not involved in disease removed were not included.

**Individual**	**SEX**	**State**	**A**	**B**	**C**	**D**	**E**	**F**	**G**	**H**	**I**	**J**	**K**
**301**	M	MR	X				X			X	X		
**302**	M	Normal IQ	X				X			X	X	X	
**303**	M	Normal IQ										X	
**304**	F	MR								X	X	X	
**307**	M	Normal IQ				X		X			X		
**309**	F	Normal IQ	X	X		X		X			X	X	
**322**	F	MR	X							X	X	X	
**323**	F	Normal IQ				X		X		X	X	X	
**324**	M	Normal IQ	X	X		X					X	X	
**310**	M	Normal IQ	X				X	X		X			
**311**	M	Normal IQ	X			X	X			X		X	X
**330**	M	Normal IQ	X				X	X		X	X	X	
**829**	F	MR	X				X	X		X	X	X	
**830**	M	MR	X								X		X
**319**	F	MR	X		X			X		X		X	
**321**	F	Normal IQ	X		X	X		X	X	X	X	X	
**u1**	M	MR	X						X	X		X	
**u2**	M	MR	X						X	X		X	X
**u3**	M	MR	X					X		X	X	X	X
**329**	M	MR	X				X			X		X	

**Table 2:** Copy number variants encountered in the Northern Swedish family: Description of the aberrations. Aberrations found only once, and in healthy individuals, and aberrations apparently not involved in disease removed were not included.

**Aberr.**	**Chrom**	**Pos. [Mb]**	**Signal**
**A**	4	154	> 3
**B**	6	161	3
**C**	8	12,4	2,5
**D**	9	31,7	2,5
**E**	10	47	2,5
**F**	11	19	3
**G**	11	50–55	3
**H**	14	0–20	2,5
**I**	14	106	3
**J**	15	0–20	2,5
**K**	22	24	2,5

## Discussion

The present study is a follow-up of a previously report by Gustavson *et al *in 1986, who described a large family from a Northern Swedish isolate with an apparently autosomal recessive form of MR [15]. Clinical, biochemical and genetic examinations have been extensive but inconsistent. Large families in geographic isolates, like the one presented in this report, have represented an important and powerful tool in genetically mapping inherited disorders [27]. We conducted a series of genetic studies in this pedigree, aiming to identify a gene responsible for this form of non-syndromic mental retardation.

Genome wide screen linkage analysis using in 500 polymorphic microsatellite markers, showed no significant LOD scores. Several peaks between 1 and 2 were encountered using both parametric LOD score analysis, and with MOD score analysis, particularly at chromosomes 13, 15 and 17, which replicate with two different algorithms (See additional file 5 to visualise these loci). Copy number analysis did not reveal a clear genomic cause for the disorder, such as a copy number alteration that would be present only in the affected individuals [28,29]. Instead, the analysis showed several variants in the family members, irrespectively of their affected status. Interestingly, de Vries *et al *in 2005 [30] have evidenced that most of the copy number changes found in patients are familiar; and therefore thought to be polymorphisms. Indeed, the issue of deciding whether or not a copy number change is pathological or polymorphic can be challenging in complex traits, such seems to be the case in this form of MR, where the number of single genes involved can be so high. Furthermore, several lines of evidence have indicated that copy number variations may be more common in the population than previously though; and they may represent normal variants, some of which may influence the risk to diseases [31,32].

These results suggest that mental retardation in this family, unlikely what we were expecting, has a heterogeneous aetiology. It may be induced by several lower effect genes variants, probably including copy number variants, which may affect neurodevelopmental genes known to exist in the regions indicated by this study. The ability to detect low risk effect genetic variants requires large samples, which may be the reason why we didn't find a significant result.

These results were not totally surprising. It has previously been demonstrated, in a twin study, that milder forms of MR have a heritability of about 50%, suggesting that half of the phenotypic variation of intelligence is explained by genetic variation [33]. This finding supports the quantitative trait loci (QTLs) hypothesis that mild MR is caused by the same multiple genes that operate throughout the distribution of general intelligence. It is possible that many quantitative trait loci for complex traits such as the general intelligence are of similarly small effect size [34]. In this context, Butcher et al in 2005 have identified four risk loci (at 2q33.3, 18q22.1, 6q25.3 and 7q11.21) [35] and a possible QTL in the heat-shock cognate protein 8 gene (HSPA8), on the long arm of chromosome 11 [36], of modest but significant effect, associated with mild mental impairment in large cohorts. The authors emphasize that mild mental retardation should not be assumed to be due to rare single-gene or chromosomal causes, but rather caused by the multiple genes that operate throughout the normal bell-shaped distribution of cognitive ability. Our results support this hypothesis [37,38].

## Conclusion

This report presents a form of MR of complex aetiology in a large isolated pedigree, where genetic variants may underlie the risk to this neurodevelopmental disorder. The geographical isolation may have favoured the founder effect and the pooling of genetic risk factors in this population, which is characterized by low genetic variation, as well as the skewing of selective influences.

Unable to identify a gene, this study indicates that the ascertainment of the cause of MR may be challenging and that a complex aetiology may be present even in a consanguineous family, constituting an additional obstacle for genetic counselling. Variants in genes involved in molecular mechanisms of cellular plasticity, in genes involved in the development of underlying neural architectures, and in genes involved in neurodevelopment and in the ongoing function of terminally differentiated neurons may underlie the phenotypic variation of intelligence and explain instances of intellectual impairment.

## Competing interests

The author(s) declare that they have no competing interests.

## Authors' contributions

ABM planned and carried out most of the molecular genetic studies, most of the statistical analysis and drafted the manuscript.

IK supervised, coordinated and participated in the statistical analysis efforts.

QPY carried out the initial family study and conducted the initial genotyping.

SP participated in the statistical analysis, and supervised ABM with the use of SimWalk.

LW participated in the design of the study and patient recruitment.

KHG examined the patients, conceived the study and participated in its design and coordination.

MS coordinated the genetic analysis on the study and participated in its design and coordination.

All authors read and approved the final manuscript

## Pre-publication history

The pre-publication history for this paper can be accessed here:



## Supplementary Material

Additional file 1 supl1simwalk. MODSCORE analysis. Click here for file

Additional file 2 Supplement 2. Copy Number Variation analysis. Individuals with mental retardation are marked with MR before their codes, to facilitate the analysis. Click here for file

Additional file 3 Supplement 3. Copy number variants present in more than 5 individuals. Click here for file

Additional file 4 Supplement 4. Copy number variants present only in patients or only in controls. Click here for file

Additional file 5 Supplement 5. Chromosomes highlighted by the linkage analysis. Click here for file
