# Supplementary figures and images for: Complex aetiology of an apparently Mendelian form of Mental Retardation
Source: BMC Med Genet. 2008 Feb 6;9:6. doi: 10.1186/1471-2350-9-6 (PMC2259315; doi:10.1186/1471-2350-9-6)

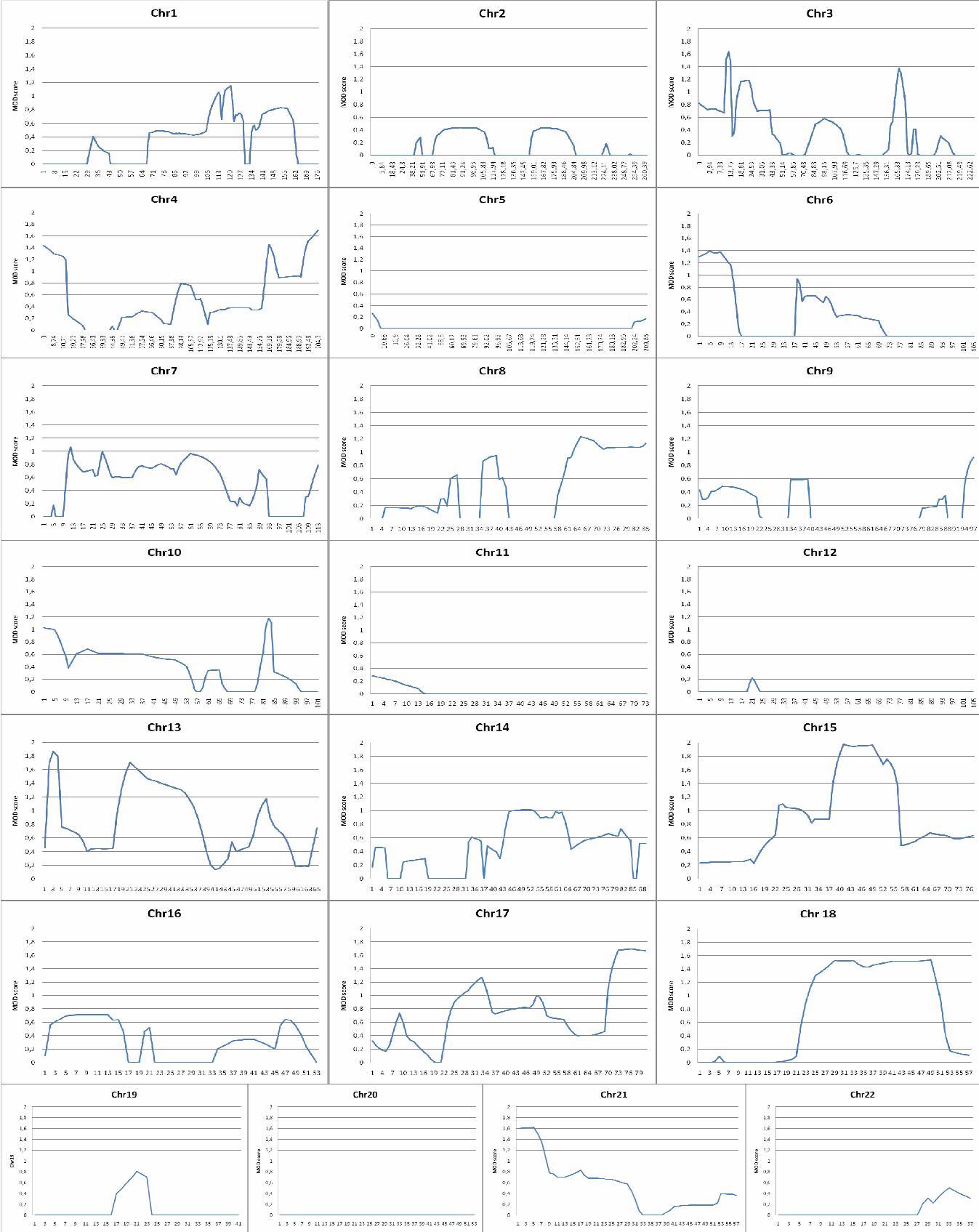

Supplement: Additional file 1 — supl1simwalk. MODSCORE analysis. [file 1471-2350-9-6-S1.jpeg]

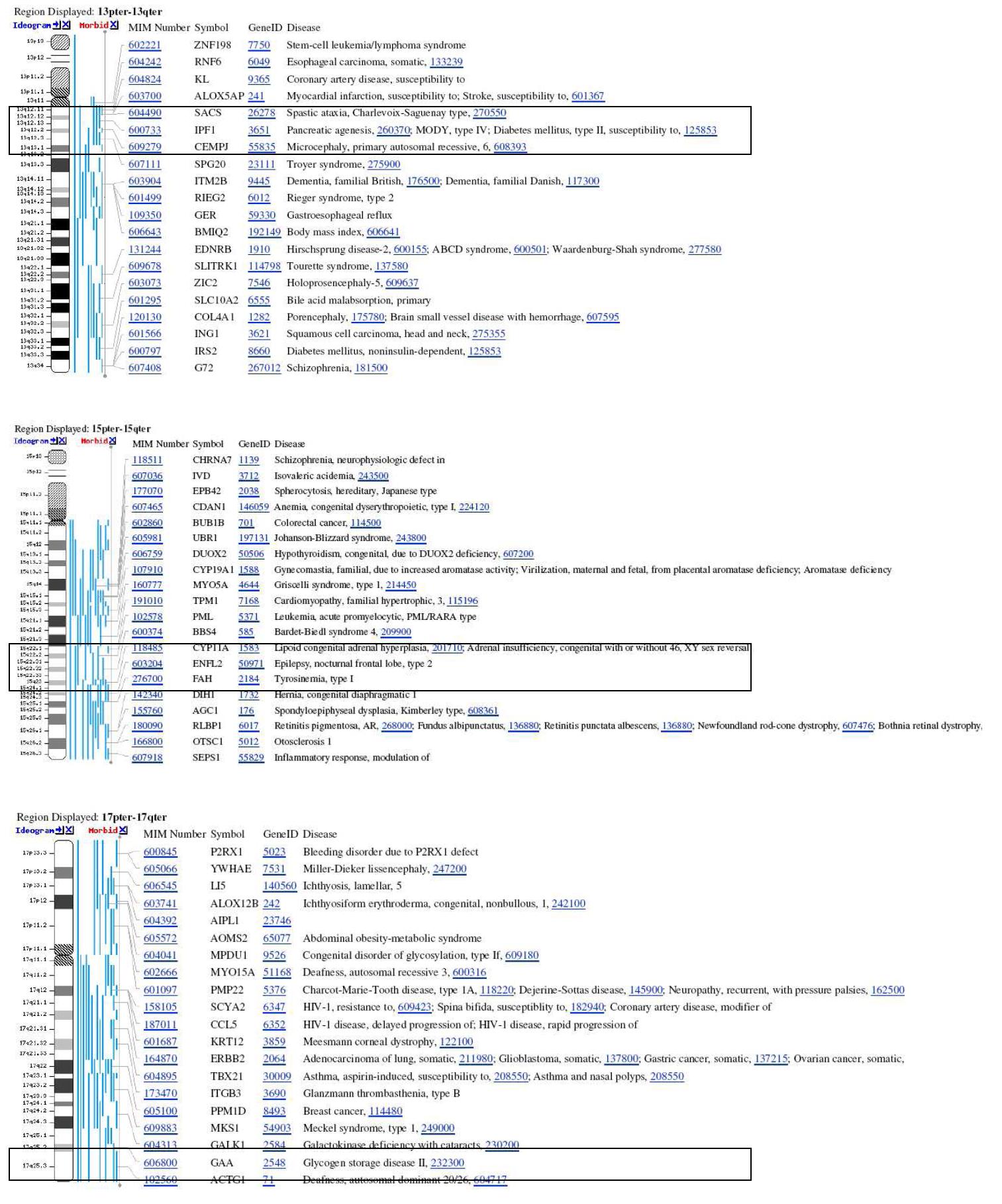

Supplement: Additional file 5 — Supplement 5. Chromosomes highlighted by the linkage analysis. [file 1471-2350-9-6-S5.jpeg]
