# Supplementary material for: Complex aetiology of an apparently Mendelian form of Mental Retardation
Source: BMC Med Genet. 2008 Feb 6;9:6. doi: 10.1186/1471-2350-9-6 (PMC2259315; doi:10.1186/1471-2350-9-6)
Supplement: Additional file 2 — Supplement 2. Copy Number Variation analysis. Individuals with mental retardation are marked with MR before their codes, to facilitate the analysis. [file 1471-2350-9-6-S2.doc]

Supplement 2: Copy Number Variation analysis. Individuals with mental retardation are marked with MR before their codes, to facilitate the analysis.

| **Signal** | **Chr** | **Start position** | **Stop position** | **Sample,IDs,in,composite,island** |
| --- | --- | --- | --- | --- |
| **3** | 1 | 2137143 | 2585920 | MR829,330,SZ329,323,MR322,321,302,MRu3 |
| **1** | 1 | 12240880 | 12509356 | 324,307 |
| **1** | 1 | 16668122 | 16790952 | 302 |
| **3** | 1 | 16689150 | 16790952 | MRu2,MRu1,321,MR319,307 |
| **3** | 1 | 21343247 | 21488043 | 330,SZ329 |
| **1** | 1 | 32223678 | 32290332 | 307 |
| **1** | 1 | 32946173 | 33179363 | MRu2,MR829 |
| **3** | 1 | 35267207 | 35356987 | MR322 |
| **1** | 1 | 42720837 | 42884271 | 309 |
| **1** | 1 | 46174146 | 46320677 | MR830,MRu3 |
| **1** | 1 | 51616526 | 51676726 | MR830 |
| **3** | 1 | 73131678 | 73387357 | 311,307 |
| **3** | 1 | 103779021 | 103840794 | MRu1,324,323,321,310,303,302,MR301,MRu3 |
| **1** | 1 | 141501392 | 141508099 | 311 |
| **3** | 1 | 145700996 | 146524632 | MRu2,MRu1,330,SZ329,324,MR322,321,MR319,307,303,MR301 |
| **1** | 1 | 151880356 | 151943464 | MR830,MR829,311 |
| **3** | 1 | 152465154 | 152623540 | MR322 |
| **3** | 1 | 158001966 | 158090820 | 324 |
| **1** | 1 | 231667051 | 231701860 | 307 |
| **1** | 2 | 66307057 | 66483118 | 323 |
| **3** | 2 | 83327506 | 83462730 | 324 |
| **3** | 2 | 87402389 | 87811112 | MRu1 |
| **3** | 2 | 89125032 | 89453770 | 330 |
| **3** | 2 | 89772948 | 89808862 | MR301 |
| **1** | 2 | 94972832 | 95037031 | MR830,MR829,324,MR319,311,310,MRu3 |
| **3** | 2 | 95661647 | 95792445 | MR830,MR829,MR322,MR319,307 |
| **1** | 2 | 112118448 | 112158449 | MR322,MR319,309 |
| **1** | 2 | 130758663 | 130859366 | MRu1,SZ329,321,MR319 |
| **1** | 2 | 131931874 | 132072532 | MRu2,MR319 |
| **3** | 2 | 165178665 | 165316732 | 309,MRu3 |
| **3** | 2 | 203295094 | 203395993 | MR322,311,303 |
| **3** | 2 | 241162716 | 241316775 | 321 |
| **1** | 3 | 16823923 | 17053844 | 330,311 |
| **1** | 3 | 17667819 | 17777885 | MRu3 |
| **1** | 3 | 48702116 | 48947355 | MRu2 |
| **3** | 3 | 52183938 | 52421828 | 323,MR322,MR319,302,MR301,MRu3 |
| **1** | 3 | 75460405 | 75691178 | MRu3 |
| **3** | 3 | 75627862 | 76043908 | MRu1,330,302,MR301 |
| **3** | 3 | 79310242 | 79531884 | MRu3 |
| **3** | 3 | 84811104 | 84964169 | 307 |
| **3** | 3 | 95031169 | 95150460 | 307 |
| **3** | 3 | 97476264 | 97628365 | MRu1,307 |
| **1** | 3 | 196743569 | 197016536 | MRu1,SZ329,321 |
| **1** | 3 | 197920477 | 198008188 | 310 |
| **3** | 4 | 19099 | 211623 | 330,303,302 |
| **3** | 4 | 3542417 | 3751308 | MRu2,330,321 |
| **3** | 4 | 9058087 | 9128192 | 323,MR322 |
| **3** | 4 | 32532226 | 32721379 | 311 |
| **3** | 4 | 39525760 | 39758431 | 323,321,MR319 |
| **3** | 4 | 69213372 | 69318438 | MR830,MR829,330,323,321,MR319,310,309,307,303 |
| **4** | 4 | 69213372 | 69318438 | MRu2,MRu1,SZ329,324,311,302,MR301 |
| **3** | 4 | 123596569 | 123752646 | 311,302 |
| **3** | 4 | 127263004 | 127571508 | 311 |
| **1** | 4 | 161293819 | 161488538 | MR301 |
| **3** | 4 | 188892738 | 189119938 | 330,307,MR301 |
| **1** | 5 | 44836422 | 45056288 | MR319,MRu3 |
| **3** | 5 | 46135310 | 46419092 | 303 |
| **1** | 5 | 68603707 | 68756175 | 324,310,309 |
| **1** | 5 | 72021135 | 72217160 | MR830 |
| **1** | 5 | 74376083 | 74499143 | 311 |
| **3** | 5 | 103099314 | 103303120 | 310 |
| **1** | 5 | 126515831 | 126670819 | 323 |
| **1** | 5 | 137257313 | 137472899 | MR829 |
| **1** | 5 | 137676403 | 137791514 | MR304 |
| **3** | 6 | 62030184 | 62153725 | MR830,307 |
| **3** | 6 | 63091649 | 63224267 | 311 |
| **1** | 6 | 79715889 | 79839756 | MR830,MR829 |
| **3** | 6 | 95985375 | 96213935 | 311 |
| **1** | 6 | 149951184 | 150175735 | 324 |
| **1** | 6 | 159098425 | 159249703 | 311 |
| **3** | 6 | 161304271 | 161524248 | 324,309,MRu3 |
| **3** | 7 | 924307 | 1089480 | 330,MR319,310 |
| **1** | 7 | 1025580 | 1089480 | 302 |
| **1** | 7 | 4874880 | 5075500 | MR830,MR829 |
| **3** | 7 | 5075500 | 5166295 | 323,MR301 |
| **1** | 7 | 44383092 | 44449526 | 324 |
| **3** | 7 | 57542879 | 62350279 | MRu2,MRu1,330,330,SZ329,MR322,311,310,309,302 |
| **4** | 7 | 61547528 | 62281083 | 302,MR301 |
| **1** | 7 | 61547528 | 61635018 | 324 |
| **1** | 7 | 65473579 | 65678735 | 302 |
| **1** | 7 | 72207080 | 72210109 | 324,31 |
| **1** | 7 | 73559909 | 73578580 | 311 |
| **3** | 7 | 76062226 | 76210401 | MRu1 |
| **1** | 7 | 91229007 | 91454308 | MR830,MR829,321,310 |
| **1** | 7 | 99138462 | 99287396 | MRu3 |
| **3** | 7 | 100944225 | 101048051 | MR322 |
| **3** | 7 | 101614079 | 101658775 | 323 |
| **3** | 7 | 138181122 | 138266702 | 323 |
| **1** | 7 | 138450431 | 138579834 | 309 |
| **3** | 7 | 156416675 | 156578380 | 330 |
| **1** | 7 | 158253724 | 158605053 | 307 |
| **4** | 8 | 7222992 | 7278542 | MRu1,MR829,330,321,MR319,311,303 |
| **3** | 8 | 7222992 | 7278542 | MRu2,MR830,MR322,310,MR304,302 |
| **3** | 8 | 12285367 | 12526350 | 330,321,303,302 |
| **4** | 8 | 12285367 | 12466379 | MRu2,MRu1,MR319 |
| **1** | 8 | 12285367 | 12286403 | SZ329,323 |
| **3** | 8 | 35875206 | 36340552 | 330,SZ329,311,310 |
| **1** | 8 | 42004277 | 42502241 | 309 |
| **3** | 8 | 43425303 | 47165297 | 302 |
| **1** | 8 | 48112750 | 48221951 | MR304 |
| **1** | 8 | 48489083 | 48723259 | 302 |
| **1** | 8 | 55225990 | 55286628 | 311 |
| **1** | 8 | 92240396 | 92482498 | MR322 |
| **3** | 8 | 112560844 | 112901422 | MRu2,MRu1 |
| **1** | 9 | 42937560 | 42960665 | MR829,323,302 |
| **3** | 9 | 42937560 | 44108554 | SZ329,307 |
| **1** | 9 | 43847424 | 43863873 | 324,MR301 |
| **1** | 9 | 66714351 | 67354917 | 323,MRu3 |
| **3** | 9 | 92554907 | 92804105 | MR322,321 |
| **1** | 9 | 113480490 | 113633175 | MR829 |
| **1** | 9 | 124813480 | 124891685 | 303 |
| **1** | 9 | 128730758 | 128779763 | MRu2,311 |
| **3** | 9 | 134286950 | 134446258 | MR830,MR304,MR301 |
| **3** | 10 | 46363383 | 47154881 | MR829,330,SZ329,324,311,310,302,MR301 |
| **1** | 10 | 47030119 | 47154881 | 307,MR304 |
| **1** | 10 | 102836500 | 103036358 | 311,31 |
| **3** | 10 | 104838897 | 105035718 | MR322 |
| **1** | 11 | 201447 | 430343 | 311 |
| **3** | 11 | 662443 | 745659 | MR322,MR301 |
| **1** | 11 | 932344 | 1101474 | MRu1 |
| **1** | 11 | 18850912 | 18952240 | 302,MR301 |
| **3** | 11 | 47263161 | 47461018 | 330,321 |
| **3** | 11 | 51061717 | 54592211 | MRu2,MRu1,MR830,MR829,SZ329,324,MR322,311,309,307,MR304,MR301 |
| **4** | 11 | 54592199 | 55684167 | MRu2,MRu1,321 |
| **3** | 11 | 55723902 | 55847156 | MRu2,MRu1,321 |
| **3** | 11 | 66917537 | 67014132 | MR322 |
| **3** | 11 | 99748186 | 99850648 | 330 |
| **3** | 12 | 6879531 | 6982497 | MR322 |
| **1** | 12 | 7147353 | 7346864 | 307 |
| **3** | 12 | 33778766 | 34359792 | MR830,324,311,310,309,307 |
| **3** | 12 | 36144018 | 36274326 | 302 |
| **1** | 12 | 82091386 | 83603481 | MR829,33 |
| **1** | 12 | 121816386 | 121972808 | MRu2 |
| **1** | 12 | 122107735 | 122347383 | MR829,310,MRu3 |
| **3** | 13 | 56144428 | 56241609 | MRu2,MRu1,SZ329,321,311,307 |
| **3** | 13 | 82590845 | 82728487 | 307 |
| **3** | 13 | 85897841 | 86066629 | 324 |
| **3** | 13 | 112444815 | 112612617 | 323 |
| **1** | 14 | 19336854 | 19556947 | MR830,324,309,307,303 |
| **3** | 14 | 19336854 | 19456378 | SZ329,MR322,MR301 |
| **1** | 14 | 21525454 | 21960251 | MRu2,MRu1,330,SZ329,MR322,321,MR319,311,307,MR304,303,MR301,MRu3 |
| **3** | 14 | 101366968 | 102003100 | MR322,MR319 |
| **1** | 14 | 102223944 | 102481588 | 309 |
| **3** | 14 | 105042939 | 106356482 | MRu2,MRu1,MR830,MR829,330,SZ329,323,MR322,MR322,MR319,311,310,MR304,303,302,MR301,MRu3 |
| **4** | 14 | 105685710 | 105829129 | 321,307 |
| **3** | 14 | 106171256 | 106356482 | MR830 |
| **3** | 15 | 18427103 | 20089383 | MRu2,MRu1,330,330,SZ329,323,MR322,321,MR319,311,311,309,303 |
| **1** | 15 | 18711364 | 20089383 | MR830,MR829,324,310,MR301 |
| **1** | 15 | 18758300 | 19407629 | 310,307,303 |
| **4** | 15 | 19127807 | 19407629 | 330,311 |
| **3** | 15 | 39633309 | 39777218 | 321,MR319 |
| **1** | 15 | 41547066 | 41717056 | MRu2 |
| **1** | 15 | 42929669 | 43037184 | 324,323,MR319,307 |
| **1** | 15 | 56793214 | 56902451 | MRu3 |
| **1** | 15 | 73479356 | 73612392 | 324,311,307,MR301 |
| **1** | 15 | 75238405 | 75590715 | MR830,MR829,324,311,309,307,MR304 |
| **1** | 15 | 81377930 | 81470710 | MR322,307 |
| **1** | 15 | 100169952 | 100192115 | 321 |
| **1** | 16 | 487298 | 592942 | 307,MRu3 |
| **3** | 16 | 2909822 | 3095007 | 330,323,321 |
| **1** | 16 | 12969112 | 13073684 | MR829,311 |
| **1** | 16 | 14993255 | 15471274 | 330,324,MR322,MR319 |
| **1** | 16 | 18514090 | 18918339 | MR830,MR829,324,323,311,309,MRu3 |
| **3** | 16 | 29122799 | 29231193 | 307 |
| **1** | 16 | 29580704 | 30085308 | 311,MR301,MRu3 |
| **3** | 16 | 30085308 | 30619067 | MR830,323,MR322 |
| **1** | 16 | 30619067 | 30879311 | MRu2,MRu1 |
| **3** | 16 | 32370399 | 32818585 | MRu1 |
| **3** | 16 | 34947701 | 34996986 | MR322,321,311,307,MR301,MRu3 |
| **1** | 16 | 45956961 | 46078672 | MR304 |
| **3** | 16 | 63008878 | 63189754 | MR322 |
| **1** | 16 | 66144782 | 66163295 | 307 |
| **3** | 16 | 83528924 | 83656752 | MR322 |
| **3** | 16 | 86513309 | 86625612 | 330,MR322,321,MR319,302 |
| **3** | 17 | 18901 | 198273 | 330,SZ329 |
| **3** | 17 | 2555238 | 2757422 | SZ329,MR322,321 |
| **3** | 17 | 7214801 | 7580474 | MR830,330,SZ329,MR322 |
| **3** | 17 | 24749129 | 24836351 | MR319 |
| **1** | 17 | 26238513 | 26602486 | MR829,310,309 |
| **3** | 17 | 35542587 | 35677005 | 330 |
| **3** | 17 | 40815480 | 40897617 | 323 |
| **3** | 17 | 41513416 | 41719833 | MRu2,323,MR322,MR319 |
| **1** | 17 | 53540080 | 53617537 | 307 |
| **3** | 17 | 57672361 | 57845878 | 311 |
| **1** | 17 | 64593327 | 64772833 | 323 |
| **1** | 17 | 70773888 | 71158961 | 309,307 |
| **3** | 17 | 77142551 | 77303541 | 323 |
| **3** | 18 | 14336307 | 14342852 | MR830 |
| **1** | 18 | 15039329 | 15096727 | MR319,307,MR304,303 |
| **3** | 19 | 212033 | 2192327 | 330,323,MR322,321,MR319 |
| **3** | 19 | 5032364 | 5325532 | 330 |
| **1** | 19 | 6217778 | 6353648 | MRu2,303 |
| **3** | 19 | 7442221 | 7568529 | 323 |
| **3** | 19 | 9976345 | 10171392 | 324 |
| **1** | 19 | 12502060 | 12578847 | 311 |
| **1** | 19 | 12782186 | 13016709 | 310,303,302 |
| **3** | 19 | 12782186 | 12811948 | 321 |
| **3** | 19 | 16931679 | 17144942 | 330,MR301 |
| **3** | 19 | 20449621 | 20535101 | MRu2 |
| **3** | 19 | 21794451 | 22109599 | 330 |
| **1** | 19 | 32651846 | 32761177 | 323 |
| **3** | 19 | 42365573 | 42515651 | 323 |
| **3** | 19 | 47368321 | 47398150 | 323 |
| **3** | 19 | 48024719 | 48230781 | MRu2,303 |
| **3** | 19 | 50021054 | 50382729 | 323 |
| **3** | 19 | 50674620 | 50850313 | MR322 |
| **1** | 19 | 52210724 | 52458325 | 311 |
| **3** | 19 | 52823629 | 52953730 | 330 |
| **1** | 19 | 53643691 | 53777545 | 311 |
| **3** | 19 | 53999660 | 54282750 | 330,MR322,MR301 |
| **1** | 19 | 54606508 | 54973181 | 307 |
| **3** | 19 | 55184866 | 55255510 | 302 |
| **3** | 19 | 59217568 | 59506905 | 323,MR322 |
| **1** | 20 | 28106854 | 28119554 | 311,307,MR304,303,MR301 |
| **3** | 20 | 28106854 | 28119554 | 321 |
| **3** | 20 | 61628090 | 61879921 | 330,321 |
| **1** | 21 | 9887804 | 13291078 | 309 |
| **3** | 21 | 9887804 | 9984282 | 303 |
| **3** | 21 | 13517135 | 13741579 | MRu2,MRu1 |
| **1** | 22 | 14441016 | 14490036 | MR830,324,321,311 |
| **1** | 22 | 17269781 | 17460737 | MR830,323,310 |
| **3** | 22 | 19682156 | 19786161 | MR322 |
| **3** | 22 | 20667608 | 20982454 | MRu3 |
| **3** | 22 | 21169794 | 21482039 | MRu2,323,MR319,302 |
| **3** | 22 | 24043842 | 24287542 | MRu2,MR830,311 |
| **3** | 22 | 40083576 | 40174013 | 330 |
| **3** | 22 | 41243357 | 41397529 | MR322 |
