# Supplementary material for: Complex aetiology of an apparently Mendelian form of Mental Retardation
Source: BMC Med Genet. 2008 Feb 6;9:6. doi: 10.1186/1471-2350-9-6 (PMC2259315; doi:10.1186/1471-2350-9-6)
Supplement: Additional file 3 — Supplement 3. Copy number variants present in more than 5 individuals. [file 1471-2350-9-6-S3.doc]

Supplement 3: Copy number variants present in more than 5 individuals.

| **CN** | **Chr** | **Start pos** | **Stop pos** | **Sample IDs** |
| --- | --- | --- | --- | --- |
| 3 | 1 | 2137143 | 2585920 | MR829,330,SZ329,323,MR322,321,302,MRu3 |
| 3 | 1 | 16689150 | 16790952 | MRu2,MRu1,321,MR319,307 |
| 3 | 1 | 103779021 | 103840794 | MRu1,324,323,321,310,303,302,MR301,MRu3 |
| 3 | 1 | 145700996 | 146524632 | MRu2,MRu1,330,SZ329,324,MR322,321,MR319,307,303,MR301 |
| 1 | 2 | 94972832 | 95037031 | MR830,MR829,324,MR319,311,310,MRu3 |
| 3 | 2 | 95661647 | 95792445 | MR830,MR829,MR322,MR319,307 |
| 3 | 3 | 52183938 | 52421828 | 323,MR322,MR319,302,MR301,MRu3 |
| 3 | 4 | 69213372 | 69318438 | MR830,MR829,330,323,321,MR319,310,309,307,303 |
| 4 | 4 | 69213372 | 69318438 | MRu2,MRu1,SZ329,324,311,302,MR301 |
| 3 | 7 | 57542879 | 62350279 | MRu2,MRu1,330,330,SZ329,MR322,311,310,309,302 |
| 4 | 8 | 7222992 | 7278542 | MRu1,MR829,330,321,MR319,311,303 |
| 3 | 8 | 7222992 | 7278542 | MRu2,MR830,MR322,310,MR304,302 |
| 3 | 10 | 46363383 | 47154881 | MR829,330,SZ329,324,311,310,302,MR301 |
| 3 | 11 | 51061717 | 54592211 | MRu2,MRu1,MR830,MR829,SZ329,324,MR322,311,309,307,MR304,MR301 |
| 3 | 12 | 33778766 | 34359792 | MR830,324,311,310,309,307 |
| 3 | 13 | 56144428 | 56241609 | MRu2,MRu1,SZ329,321,311,307 |
| 1 | 14 | 19336854 | 19556947 | MR830,324,309,307,303 |
| 1 | 14 | 21525454 | 21960251 | MRu2,MRu1,330,SZ329,MR322,321,MR319,311,307,MR304,303,MR301,MRu3 |
| 3 | 14 | 105042939 | 106356482 | MRu2,MRu1,MR830,MR829,330,SZ329,323,MR322,MR322,MR319,311,310,MR304,303,302,MR301,MRu3 |
| 3 | 15 | 18427103 | 20089383 | MRu2,MRu1,330,330,SZ329,323,MR322,321,MR319,311,311,309,303 |
| 1 | 15 | 18711364 | 20089383 | MR830,MR829,324,310,MR301 |
| 1 | 15 | 75238405 | 75590715 | MR830,MR829,324,311,309,307,MR304 |
| 1 | 16 | 18514090 | 18918339 | MR830,MR829,324,323,311,309,MRu3 |
| 3 | 16 | 34947701 | 34996986 | MR322,321,311,307,MR301,MRu3 |
| 3 | 16 | 86513309 | 86625612 | 330,MR322,321,MR319,302 |
| 3 | 19 | 212033 | 2192327 | 330,323,MR322,321,MR319 |
| 1 | 20 | 28106854 | 28119554 | 311,307,MR304,303,MR301 |
