# Supplementary material for: Complex aetiology of an apparently Mendelian form of Mental Retardation
Source: BMC Med Genet. 2008 Feb 6;9:6. doi: 10.1186/1471-2350-9-6 (PMC2259315; doi:10.1186/1471-2350-9-6)
Supplement: Additional file 4 — Supplement 4. Copy number variants present only in patients or only in controls. [file 1471-2350-9-6-S4.doc]

Supplement 4: Copy number variants present only in patients or only in controls

| **CN** | **Chr** | **Start pos** | **Stop pos** | **Sample IDs** |
| --- | --- | --- | --- | --- |
| 1 | 1 | 32946173 | 33179363 | MRu2,MR829 |
| 1 | 1 | 46174146 | 46320677 | MR830,MRu3 |
| 3 | 1 | 73131678 | 73387357 | 311,307 |
| 1 | 2 | 131931874 | 132072532 | MRu2,MR319 |
| 3 | 2 | 165178665 | 165316732 | 309,MRu3 |
| 3 | 2 | 203295094 | 203395993 | MR322,311,303 |
| 1 | 3 | 16823923 | 17053844 | 330,311 |
| 3 | 4 | 19099 | 211623 | 330,303,302 |
| 1 | 5 | 68603707 | 68756175 | 324,310,309 |
| 1 | 6 | 79715889 | 79839756 | MR830,MR829 |
| 1 | 7 | 4874880 | 5075500 | MR830,MR829 |
| 4 | 7 | 61547528 | 62281083 | 302,MR301 |
| 1 | 7 | 72207080 | 72210109 | 324,31 |
| 4 | 8 | 12285367 | 12466379 | MRu2,MRu1,MR319 |
| 3 | 8 | 35875206 | 36340552 | 330,SZ329,311,310 |
| 3 | 8 | 112560844 | 112901422 | MRu2,MRu1 |
| 3 | 9 | 134286950 | 134446258 | MR830,MR304,MR301 |
| 3 | 11 | 662443 | 745659 | MR322,MR301 |
| 3 | 11 | 47263161 | 47461018 | 330,321 |
| 3 | 14 | 19336854 | 19456378 | SZ329,MR322,MR301 |
| 3 | 14 | 101366968 | 102003100 | MR322,MR319 |
| 4 | 14 | 105685710 | 105829129 | 321,307 |
| 1 | 15 | 18758300 | 19407629 | 310,307,303 |
| 4 | 15 | 19127807 | 19407629 | 330,311 |
| 3 | 16 | 2909822 | 3095007 | 330,323,321 |
| 1 | 16 | 30619067 | 30879311 | MRu2,MRu1 |
| 1 | 17 | 70773888 | 71158961 | 309,307 |
| 3 | 18 | 14336307 | 14342852 | MR830 |
| 1 | 19 | 12782186 | 13016709 | 310,303,302 |
| 3 | 19 | 48024719 | 48230781 | MRu2,303 |
| 3 | 20 | 61628090 | 61879921 | 330,321 |
| 3 | 21 | 13517135 | 13741579 | MRu2,MRu1 |
